# The Fungal Communities and Flavor Profiles in Different Types of High-Temperature Daqu as Revealed by High-Throughput Sequencing and Electronic Senses

**DOI:** 10.3389/fmicb.2021.784651

**Published:** 2021-12-02

**Authors:** Wenchao Cai, Yu’ang Xue, Yurong Wang, Wenping Wang, Na Shu, Huijun Zhao, Fengxian Tang, Xinquan Yang, Zhuang Guo, Chunhui Shan

**Affiliations:** ^1^School of Food Science, Shihezi University, Shihezi, China; ^2^Hubei Provincial Engineering and Technology Research Center for Food Ingredients, Hubei University of Arts and Science, Xiangyang, China; ^3^Engineering Research Center for Storage and Processing of Xinjiang Characteristic Fruits and Vegetables, Ministry of Education, Shihezi University, Shihezi, China; ^4^Xiangyang Maotai-Flavor Baijiu Solid-State Fermentation Enterprise-University Joint Innovation Center, Xiangyang, China

**Keywords:** high-temperature Daqu, sauce-flavor Baijiu, fungal diversity, Illumina MiSeq high-throughput sequencing, electronic nose, electronic tongue

## Abstract

Polymicrobial co-fermentation is among the distinct character of high-temperature Daqu. However, fungal communities in the three types of high-temperature Daqu, namely, white high-temperature Daqu, black high-temperature Daqu, and yellow high-temperature Daqu, are yet to be characterized. In this study, the fungal diversity, taste, and aroma profiles in the three types of high-temperature Daqu were investigated by Illumina MiSeq high-throughput sequencing, electronic tongue, and electronic nose, respectively. Ascomycota and Basidiomycota were detected as the absolute dominant fungal phylum in all types of high-temperature Daqu samples, whereas *Thermomyces*, *Thermoascus*, *Aspergillus*, *Rasamsonia*, *Byssochlamys*, and *Trichomonascus* were identified as the dominant fungal genera. The fungal communities of the three types of high-temperature Daqu differed significantly (*p* < 0.05), and *Thermomyces*, *Thermoascus*, and *Monascus* could serve as the biomarkers in white high-temperature Daqu, black high-temperature Daqu, and yellow high-temperature Daqu, respectively. The three types of high-temperature Daqu had an extremely significant difference (*p* < 0.01) in flavor: white high-temperature Daqu was characterized by sourness, bitterness, astringency, richness, methane, alcohols, ketones, nitrogen oxides, and sulfur organic compounds; black high-temperature Daqu was characterized by aftertaste-A, aftertaste-B, methane-aliph, hydrogen, and aromatic compounds; and yellow high-temperature Daqu was characterized by saltiness, umami, methane, alcohols, ketones, nitrogen oxides, and sulfur organic compounds. The fungal communities in the three types of high-temperature Daqu were significantly correlated with taste but not with aroma, and the aroma of high-temperature Daqu was mainly influenced by the dominant fungal genera including *Trichomonascus*, *Aspergillus*, *Thermoascus*, and *Thermomyces*. The result of the present study enriched and refined our knowledge of high-temperature Daqu, which had positive implications for the development of traditional brewing technique.

## Introduction

Chinese Baijiu is a typical Chinese traditional fermented alcoholic beverage and is one of the best known distilled liquors in the world (Xu and Ji, 2011; Huang et al., 2017; Wang et al., 2017). Of them, sauce-flavor Baijiu (SFB) is among the three classic flavor-type Chinese Baijiu with the most complex brewing process. It is welcomed by consumers, and its unique flavor characteristics are mainly derived from its special year-long process with several complicated steps of three stages: the Daqu (starter) making, stacking fermentation on the ground, and alcoholic fermentation in the pits (Dai et al., 2020; Wang H. et al., 2021). Specifically, a mixture of crushed and steamed grain (sorghum) and mature high-temperature Daqu (HTD) is piled and fermented on the ground for 3–7 days (the process is known as stacking fermentation) (Chen et al., 2014; Zhang et al., 2021). The mixture is subsequently transferred into a pit and further fermented for another 30 days (Yan et al., 2020). It is then removed from the pit and distilled into raw SFB (Wang H. et al., 2021). This complex solid-state fermentation process is repeated for seven cycles in an open and uncontrolled environment with multi-microorganisms (Li et al., 2016; Du et al., 2019; Liu et al., 2019). These microorganisms have a major impact on fermentation yield and quality, mainly derived from HTD, a saccharifying starter, and raw material used in the brewing process of SFB (Li et al., 2017; Yang et al., 2018; Wang Y. R. et al., 2021). HTD is composed of pea and barley along with wheat as primary raw materials and is made by shaping, piling, fermenting, and ripening under high temperature conditions (60–70°C) (Li et al., 2016, 2017; Fan et al., 2018). Its production process can naturally collect and enrich the microorganisms in the brewing environment, forming a unique and abundant microbial community structure and thus producing rich and diverse enzymes along with flavor precursors, which has an important contribution to the Chinese Baijiu body of sauce flavor; meanwhile, it provides sufficient microbial reserves for the subsequent fermentation (Wang et al., 2008; Du et al., 2019; Liu et al., 2019; Tian et al., 2020). Therefore, the study on microbial community structure in HTD of SFB has been a hot spot in Chinese Baijiu industry. HTD has a high microbial diversity, which chiefly includes bacteria, yeasts, and molds along with tiny amounts of actinomycetes, derived from raw materials, mother Daqu (HTD produced in the last round), and production environment (Su et al., 2015; Du et al., 2019). Because the starch contained in sorghum, the main raw material for Chinese Baijiu brewing, cannot be directly utilized by most microorganisms in HTD but has to be hydrolyzed to fermentable sugars via saccharidase and alpha amylase produced by fungi, the fungi in HTD are essential in the Chinese Baijiu brewing process (Zheng X. W. et al., 2012; Chen et al., 2014). Among them, molds can secrete many kinds of hydrolytic enzymes, such as saccharidase, protease, lipase, and amylase, which can degrade starch and protein along with other macromolecular substances in raw materials and increase the content of saccharides and amino acids in the whole reaction system. This not only provides nutrients and basic substances for the growth and metabolism of other microorganisms but also conduces greatly to the subsequent formation of SFB body flavor (Endo and Okada, 2005). Yeast is an important aroma-producing fungal group during the fermentation of SFB and directly affects the yield (Wu et al., 2012). Molds and yeasts are important brewing fungi that affect the solid-state bilateral fermentation of Chinese Baijiu and ensure the normal fermentation progress, whereas the internal structure of fungi may promote each other or inhibit each other, and their different structure of fungal composition will affect the style and quality of Chinese Baijiu. Consequently, the exploration of the fungal diversity of HTD could help to comprehensively reveal their brewing microbial resources and enhance product control.

Besides, the abundant microorganisms and enzymes involved in the manufacturing process of the HTD act together in the Maillard reaction, leading to the browning of HTD and imparting sauce flavor to HTD (Gan et al., 2019; Xie et al., 2020). However, because the environmental factors such as temperature and humidity in different pile locations of HTD within the Qu room (the room where Daqu was fermented) are not consistent, the browning degree of HTD induced by Maillard reaction will also vary (Li et al., 2014). On the basis of the different colors produced after browning, the HTD was mainly classified into three types (white HTD, black HTD, and yellow HTD). White HTD is mainly located at the top of the Daqu pile, accounting for 10–15% of total HTD; black HTD is mainly located in the middle of the Daqu pile, accounting for <1% of the total HTD; whereas yellow HTD is uniformly dispersed in the Daqu pile, accounting for 80–85% of the total HTD (Gan et al., 2019; Wang Y. R. et al., 2021). During practical production, these three types of HTD are generally mixed and ground in a certain ratio for SFB fermentation (Wang Y. R. et al., 2021). For this reason, exploring the microbial diversity and flavor profiles of the three types of HTD is of great significance to Chinese Baijiu industry.

At present, study on microorganisms in HTD has focused on two aspects. First is the use of traditional molecular methods, such as culture-dependent methods or culture-independent polymerase chain reaction (PCR) denaturing gradient gel electrophoresis to screen microorganisms and apply them to production. However, this method can only screen out a part of microorganisms, and it is time-consuming with a low accuracy, so it cannot fully understand the microbial community structure in HTD (Li et al., 2014; Su et al., 2015; Huang et al., 2017; El Sheikha et al., 2018; Yang et al., 2018). Second is the application of high-throughput sequencing combined with bioinformatics to explore the microbial composition and comprehensively analyze the microbial diversity in HTD (Wu et al., 2017; Yang et al., 2018; El Sheikha and Hu, 2020). So far, researchers have investigated how different fermentation times (Su et al., 2015; Hu et al., 2017), different regions (Wang et al., 2017), different processing methods (Wang et al., 2019), and different environments (Li et al., 2016) affect the microbial community of HTD by using high-throughput sequencing technology. Not long ago, Wang Y. R. et al. (2021) utilized this technology in combination with Phylogenetic Investigation of Communities by Reconstruction of Unobserved States (PICRUSt) and BugBase to elucidate the bacterial diversity, function, and phenotype of white HTD, black HTD, and yellow HTD. However, to the best of our knowledge, reports on the fungal diversity of these three types of HTD have been rarely seen. Meanwhile, HTD, as one of the raw materials accounting for 50% in SFB, and its aroma and taste are also decisive indicators affecting the flavor of the final product. Therefore, the purpose of this study was to obtain an overview of the characteristic fungal communities and flavor profiles in the three types of HTD using high-throughput sequencing and electronic senses (E-senses), respectively. This will not only lay a foundation for subsequent related study but also provide a theoretical basis for the optimization of SFB brewing technology.

## Materials and Methods

### Sample Collection

The three types of HTD samples were collected from an SFB distillery in Xiangyang, Hubei Province, China, with 10 parallel samples for each type of HTD, for a total of 30 LTD samples. All HTD samples were made in the same batch and under the same conditions adopting the traditional HTD-making process. The samples were promptly ground, mixed, and pooled into sterile sampling bottles. All of the 30 HTD samples were immediately stored at −20°C until processed.

### DNA Extraction

Microbial community metagenomic DNA extraction from HTD sample (2 g) was conducted using the QIAGEN DNeasy mericon Food Kit (QIAamp DNA Microbiome Kit, QIAGEN, Inc.) in accordance with the instructions of the manufacturer. The DNA extract was checked via 1% agarose gel electrophoresis, and its quantity and quality were measured with a NanoDrop 2000 UV-vis spectrophotometry (Thermo Fisher Scientific, Inc., Wilmington, United States) (Cai et al., 2021d). Qualified DNA samples were stored in a −20°C refrigerator for use.

### Polymerase Chain Reaction Amplification and Illumina MiSeq High-Throughput Sequencing

The Internal Transcribed Spacer (ITS) regions of fungal ribosomal RNA (rRNA) gene was amplified with forward primer ITS1F (5′-CTTGGTCATTTAGAGGAAGTAA-3′) and reverse primer ITS2R (5′-GCTGCGTTCTTCATCGATGC-3′). The PCR amplification parameters of fungal rRNA gene were set as follows: 95°C for 3 min; 95°C for 30 s, 55°C for 30 s, 72°C for 45 s, 30 cycles; 72°C for 10 min. The PCR reaction mixture consisted of 4 μl of 5 × PCR buffer, 2 μl of 2.5 mMdNTPs mix, 0.8 μl of forward primer (5 μmol/L), 0.8 μl of reverse primer (5 μmol/L), 0.4 μl of DNA polymerase (5 U/μl), 10 ng of DNA template, supplemented to 20 μl with ddH_2_O.

The 30 purified DNA amplicons, diluted to a concentration of 100 nmol/L, were paired-end sequenced on a MiSeq high-throughput sequencing platform in Majorbio Bio-Pharm Technology Co., Ltd. (Shanghai, China).

### Quality Control of the Sequences

The pair-ended sequences generated through MiSeq sequencing were demultiplexed, quality-filtered, and merged using the following criteria: (i) the 300–base pair (bp) reads were truncated at any site receiving an average quality score of <20 over a 50-bp sliding window, the truncated reads shorter than 50 bp were discarded, and reads containing ambiguous characters were also discarded; (ii) only overlapping sequences longer than 10 bp were assembled according to their overlapped sequence. The maximum mismatch ratio of overlap region is 0.2. Reads that could not be assembled were discarded; and (iii) samples were distinguished according to the barcode and primers, and the sequence direction was adjusted, exact barcode matching, 2 nucleotide mismatch in primer matching.

### Bioinformatics Analysis

The primers and barcode sequences were removed from the high-quality reads via in-house Python scripts from the high-quality reads; meanwhile, all the reads were divided into different samples in the light of their barcodes. Quantitative Insights Into Microbial Ecology (QIIME) package (version 1.9.1) (Caporaso et al., 2010) was applied to perform bioinformatics analysis. The specific operational steps consult previous report (Cai et al., 2021c). Simply put, UCLUST (Edgar, 2010) was employed to classify high-quality sequences into operational taxonomic units (OTUs) at the threshold of 97% identity, whereas ChimeraSlayer (Haas et al., 2011) was used to remove potential chimeric sequences from the OUT representative set. Singleton OTUs (OTUs with only one sequence) were removed from all datasets. On the basis of the information extracted from the Greengenes (version 13.8) (DeSantis et al., 2006), Ribosomal Database Project (Release 11.5) (Cole et al., 2007), and Sliva (version 132) (Quast et al., 2013), each OTU was assigned to the lowest taxonomic level with a minimum bootstrap threshold of 80% (Cai et al., 2021a). The OTU table was subsampled correspondingly to adjust the sampling depth for all samples by the “multiple_rarefactions.py program” in the QIIME pipeline. Calculation of alpha and beta diversity was carried out according to the *de novo* taxonomic tree constructed from the representative chimera-checked OTU set by using FastTree (Price et al., 2009). OTU level-based alpha diversity indices, including observed species and Shannon diversity index, were calculated utilizing the OTU table in QIIME to assess sequence depth and bacterial diversity, respectively.

### Flavor Evaluation Using E-Senses

E-senses include electronic tongue (E-tongue) and electronic nose (E-nose). Both E-tongue and E-nose analyses were performed following the methods described by Cai et al. (2020b) with a commercial E-tongue (Taste-Sensing System SA 402B, Intelligent Sensor Technology Co., Ltd., Japan) and a Portable Electronic Nose (PEN3, Win Muster Airsense Analytics, Inc., Schwerin, Germany), respectively.

### Statistical Analysis

Principal coordinates analysis (PCoA), permutational multivariate analysis of variance (PERMANOVA) and multivariate analysis of variance (MANOVA), principal components analysis (PCA), Procrustes analysis, and correlation analysis were employed by R software (version 4.0.2^1^), whereas Linear Discriminant Analysis Effect Size (LEfSe) algorithm was applied using Python software (version 3.9.7^2^).

## Results

### Fungal α Diversity of Different Types of High-Temperature Daqu

On the basis of the extraction of metagenomic DNA from HTD samples, Illumina Miseq high-throughput sequencing technology was applied, and a dataset including 1,825,410 high-quality reads of the ITS rRNA gene were generated from 30 HTD samples, with an average of 60,847 ± 12,213 (means ± SD, range from 35,076 to 74,917) ITS rRNA gene reads per sample. At a high threshold identity cutoff level of 97% sequence similarity, 7,124 OTUs were detected. After removing singleton OTUs, the average number of OTUs per sample was 2484 1,042 ± 245 (range from 558 to 1,449). The specific sequencing information and the number statistics of each taxonomy of HTD samples were summarized in Supplementary Table 1.

Through the sparse curve (Supplementary Figure 1A) and the Shannon index curve (Supplementary Figure 1B), the existing sequencing depth was comprehensively evaluated. A comprehensive analysis of the information on fungal richness and diversity provided by the two diversity indexes depicted that (Supplementary Figure 1), even when the maximum sequencing depth of a single sample was reached, the slope of the sparse curve gradually decreased, tended to be flat but still increased (Supplementary Figure 1A), implying that new fungal species might be found in HTD samples as the sequencing depth increased. However, when the sequencing depth reached about 4,000, all Shannon index curves had approached flat and gradually entered the plateau stage (Supplementary Figure 1B), which indicates that with the expanding of the sequencing coverage, although new OTUs and fungal species might be found, the diversity of fungal microorganisms would no longer change. This suggests that the sequencing depth of this study is appropriate, and the existing sequencing quantities can well reflect the information of most fungal microorganisms in the samples. For this reason, an average of 1,825,410 ITS rRNA sequences per sample generated in this study can satisfy the demands of follow-up bioinformatics analysis (Bal et al., 2017).

**FIGURE 1:**
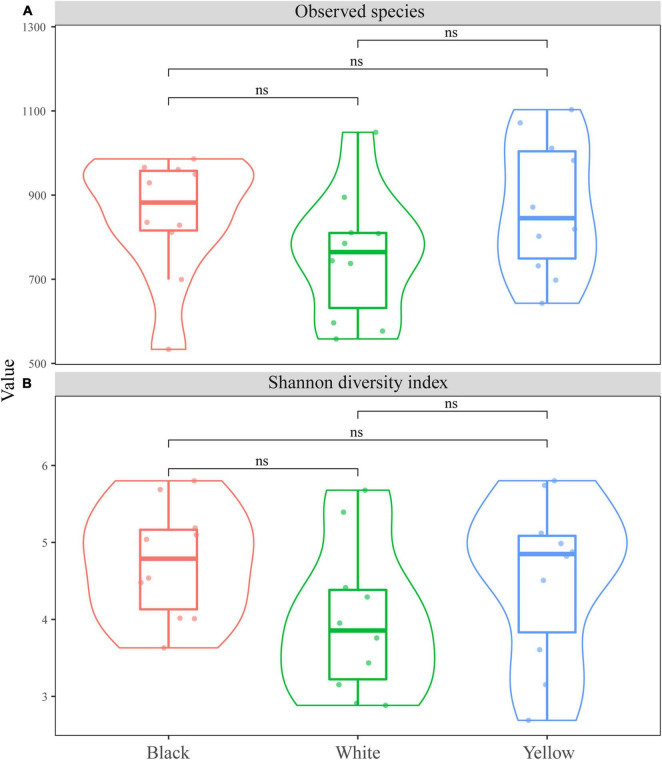
Boxplots of α-diversity indexes. **(A)** Number of observed species; **(B)** Shannon diversity index.

The fungal species richness and diversity of the three types of HTD were assessed on the basis of the number of observed species and the Shannon diversity index, respectively (Figure 1). It is observed in Figure 1 that the difference in the number of observed species and Shannon index among the three types of HTD was not significant (*p* > 0.05). This was indicative of the non-significant difference (*p* > 0.05) in both fungal richness and diversity among the different types of HTD.

### Comparison of Fungal Communities in Different Types of High-Temperature Daqu

**FIGURE 2:**
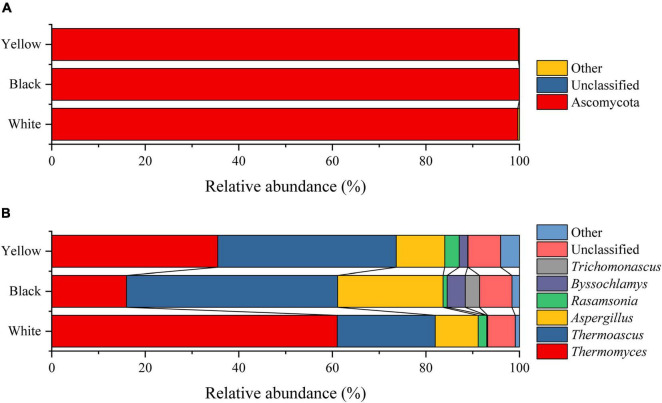
Fungal composition of HTD samples at the level of phylum **(A)** and genus **(B)**.

In the present study, homology comparisons were made on the sequences that passed the quality control, and all sequences were identified as 6 phyla, 15 classes, 26 orders, 40 families, and 54 genera. As defined by Wang Y. R. et al. (2021), the fungal phyla/genera with average relative abundances greater than 1.0% in all HTD samples were considered to be dominant phyla/genera, whereas the remaining phyla/genera with average relative abundances less than 1.00% were categorized as others. Comparative analysis in relative abundance of dominant fungal phyla and genera in the three types of HTD is presented in Figure 2.

According to the Silva database, 99.81% of the 1,825,410 optimized sequences were identified as Ascomycota (Figure 2A). Because Ascomycota was present in all HTD samples with relative abundance greater than 96.77%, it was the absolute dominant fungal phylum in HTD. At the genus level, 6 of the 54 fungal genera were the dominant fungal genera, namely, *Thermomyces* (37.49%), *Thermoascus* (34.76%), *Aspergillus* (14.04%), *Rasamsonia* (1.92%), *Byssochlamys* (1.91%), and *Trichomonascus* (1.09%), which accounted for 91.22% of the total sequences. In addition, *Thermomyces*, *Thermoascus*, and *Aspergillus* were found in all HTD samples, illustrating that they were the absolute dominant fungal genera.

**FIGURE 3:**
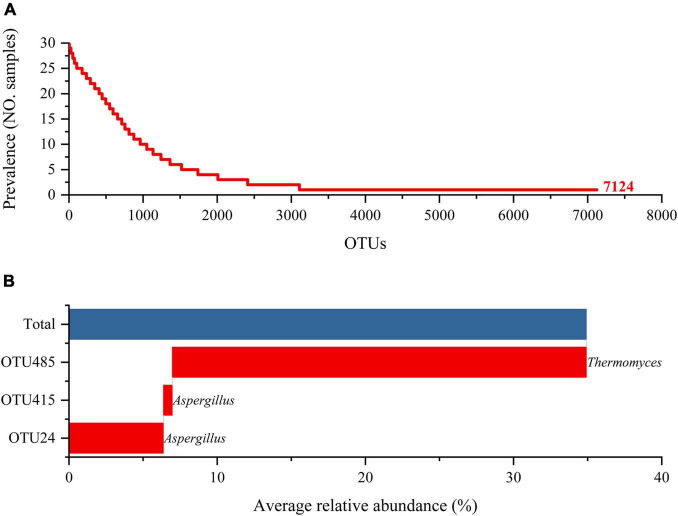
Prevalence **(A)** and average relative abundance **(B)** of OTUs in HTD samples.

Further analysis on the fungal communities in the three types of HTD samples at the OTU level was performed (Figure 3). A total of 7,124 OTUs were generated in the 30 HTD samples included in this study. Although as many as 4,016 OTUs appeared only once in the 30 samples, accounting for 56.37% of the total OTUs, the number of included sequences was only 6,911, accounting for merely 0.38% of all qualified sequences after quality control (Figure 3A). It follows that, despite that the fungal communities of the three types of HTD may contain a variety of unique fungal species, their relative abundance is extremely low.

The OTU present in each sample was considered as core OTU, and three core OTUs were obtained in all HTD samples (Figure 3B), including OTU485 (*Thermomyces*, 27.94%), OTU24 (*Aspergillus*, 6.38%), and OTU415 (*Aspergillus*, 0.60%). The relative abundance of these three core OTUs accumulated up to 34.91%, accounting for 36.63% of all qualified sequences after quality control.

### Fungal β Diversity of Different Types of High-Temperature Daqu

**FIGURE 4:**
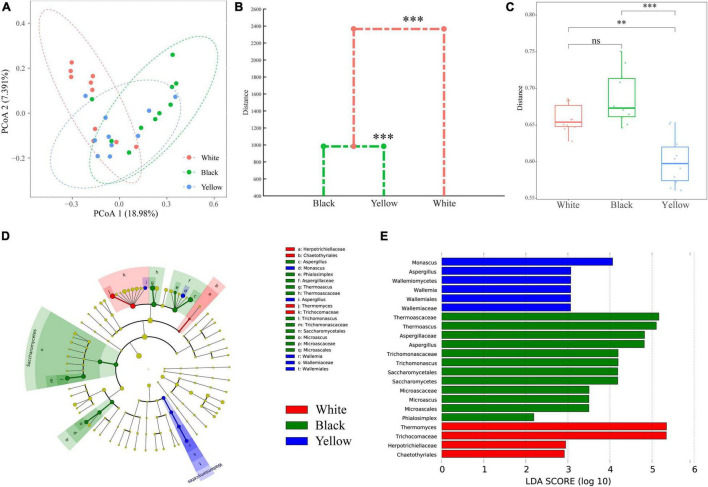
PCoA score plots based on Bray–Curtis distance **(A)**; dendrogram based on Bray–Curtis distance calculated using Mahalanobis distances and MANOVA **(B)**; within-group variations of the three types of HTD calculated on the basis of Bray–Curtis distance **(C)**. Significant difference is represented by ^***^ (0.0001 ≤ *p* < 0.001), ^**^ (0.001 ≤ *p* < 0.01), and ns (*p* ≥ 0.05), respectively. Identification of discriminant taxa among the three types of HTD by LEfSe: cladogram of the fungal communities **(D)**. Horizontal bar chart showing discriminant taxa **(E)**.

To evaluate the variation in fungal communities of the three types of HTD samples, PCoA and MANOVA on the basis of Bray–Curtis distance were employed (Figures 4A–C). It is observed in PCoA plot (Figure 4A) that black HTD samples are primarily located to the first and fourth quadrants, and white HTD samples are primarily located to the second quadrant, whereas most yellow HTD samples are located to the third and fourth quadrants. This reveals that, although there are some overlaps among the three types of HTD samples (especially on the fourth quadrant), the clustering trend is still obvious. In addition, the fungal communities of black HTD samples (mostly located to the first and fourth quadrants) and yellow HTD samples (mostly located to the third and fourth quadrants) are more similar. PERMANOVA, which measures the significance of inter- and intra-group variations (pseudo F-statistic) by permutation of group assignment, further determined the extent of compositional difference among the three types of HTD samples was extremely significant (*p* = 0.001).

Thereafter, data obtained from PCoA were assessed by a dendrogram generated from MANOVA (Figure 4B), a constrained classification of feature vectors into clusters via Mahalanobis distances, to categorize the three types of HTD samples based on Bray–Curtis distance (Cai et al., 2019, 2020a). It could be seen from Figure 4B that, among the three types of HTD samples, their fungal communities differed extremely significant (*p* < 0.001). Around the mean distance of 1,000, black HTD and yellow HTD formed a cluster; whereas around the mean distance of 2,300, white HTD, black HTD, and yellow HTD formed a cluster, demonstrating again that the fungal communities of black HTD samples and yellow HTD samples are more similar. This is also in line with PCoA.

**FIGURE 5:**
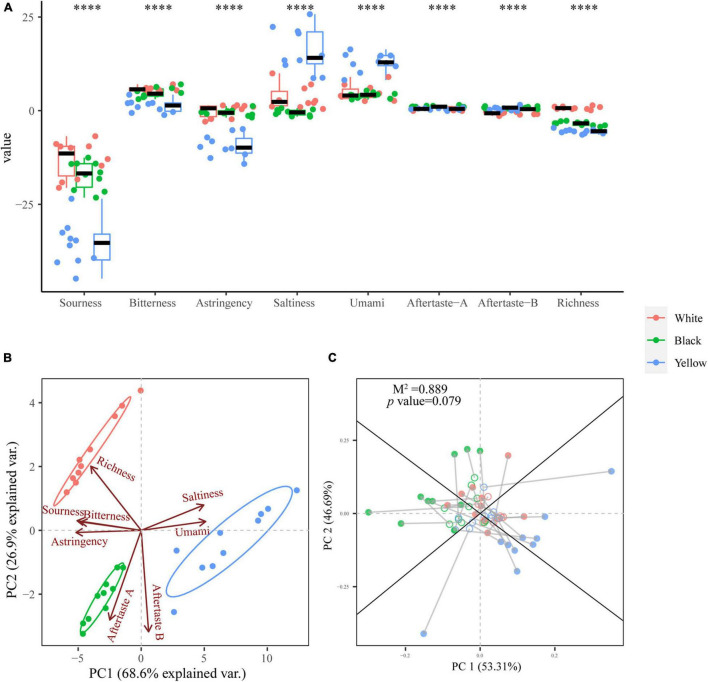
Box plot for taste profiles of the three types of HTD samples **(A)**. Significant difference is represented by ^****^ (*p* < 0.0001). PCA biplot based on the taste profiles of HTD samples **(B)**. Procrustes analysis of the correlation between dominate fungal genera and aroma profiles (*M*^2^ = 0.889, *p* = 0.079, 999 permutations) **(C)**.

Moreover, the within-group variations among the three types of HTD samples were compared (Figure 5C). Yellow HTD samples exhibited extremely significantly lower (*p* < 0.05) within-group variation than white HTD samples and black HTD samples, whereas the within-group variation between white HTD samples and black HTD samples did not differ significantly (*p* > 0.05). Besides, black HTD samples exhibited the highest within-group variation among the three types of HTD.

In summary, the fungal communities in different types of HTD samples differed significantly (*p* ≤ 0.05). LEfSe was carried out with the default linear discriminant analysis (LDA) threshold score of 2.00, to find biomarkers and identify potential distinguishable taxa in the three types of HTD (Figures 4D,E). Twenty-two differential taxa were significantly enriched (*p* < 0.05) in 30 HTD samples, of which 6, 12, and 4 were significantly enriched (*p* < 0.05) in white HTD, black HTD, and yellow HTD, respectively: The significantly enriched (*p* < 0.05) fungi in white HTD were mainly affiliated to the order Chaetothyriales, family Trichocomaceae; the significantly enriched (*p* < 0.05) fungi in black HTD were mainly affiliated to class Saccharomycetes, order Microascales, family Thermoascaceae, along with genera *Aspergillus* and *Phialosimplex*; whereas the significantly enriched (*p* < 0.05) fungi in yellow HTD were mainly affiliated to class Wallemiomycetes and genera *Monasus* and *Aspergillus*.

Furthermore, LEfSe identified biomarkers with the highest LDA values at the genus level in these three different types of HTD (Figure 4D). This indicates that *Thermomyces*, *Thermoascus*, and *Monascus* were the biomarker in white HTD, black HTD, and yellow HTD, respectively (Segata et al., 2016).

### Flavor Profiles of High-Temperature Daqu Based on Electronic Senses

The taste of HTD samples was determined using E-tongue (Figures 5A,B). As it is observed in Figure 5A, the HTD samples exhibited extremely significant differences of *p* < 0.0001 in the response values to all eight E-tongue sensors, indicating that the taste of different types of HTD differed extremely significantly (*p* < 0.0001). It is worth noting that the differences of the response values of the three types of HTD in the aftertaste-A (aftertaste of astringency) and aftertaste-B (aftertaste of bitterness) were less than 1. For a more intuitive and comprehensive understanding of the taste profiles of different types of HTD, PCA was applied to perform an unconstrained dimension reduction on the E-tongue dataset (Figure 5B). The first two principal components (PC 1 and PC2) explained 95.5% of the total variance (68.6 and 26.9%, respectively). From the PCA biplot (Figure 5B), the three different types of HTD can be clearly distinguished: Yellow HTD distributed to the first and fourth quadrants of the biplot were distinguished by saltiness and umami, whereas white and black HTD located in the second and third quadrants, respectively, were characterized primarily by sourness, bitterness, astringency, and richness. The separation of the spatial distribution of the three different types of HTD was evident with no overlap, which confirms the extremely significant difference (*p* < 0.0001) in their taste. Procrustes analysis on the basis of the PCA of both the dominant fungal genera and taste profiles of HTD samples was employed to reveal their correlation, and it turned out to be not significant (*M*^2^ = 0.889, *p* = 0.079 > 0.05) (Figure 5C). This illustrates the extremely significant divergence (*p* < 0.0001) in the taste among the three types of HTD was not caused by their fungal communities.

**FIGURE 6:**
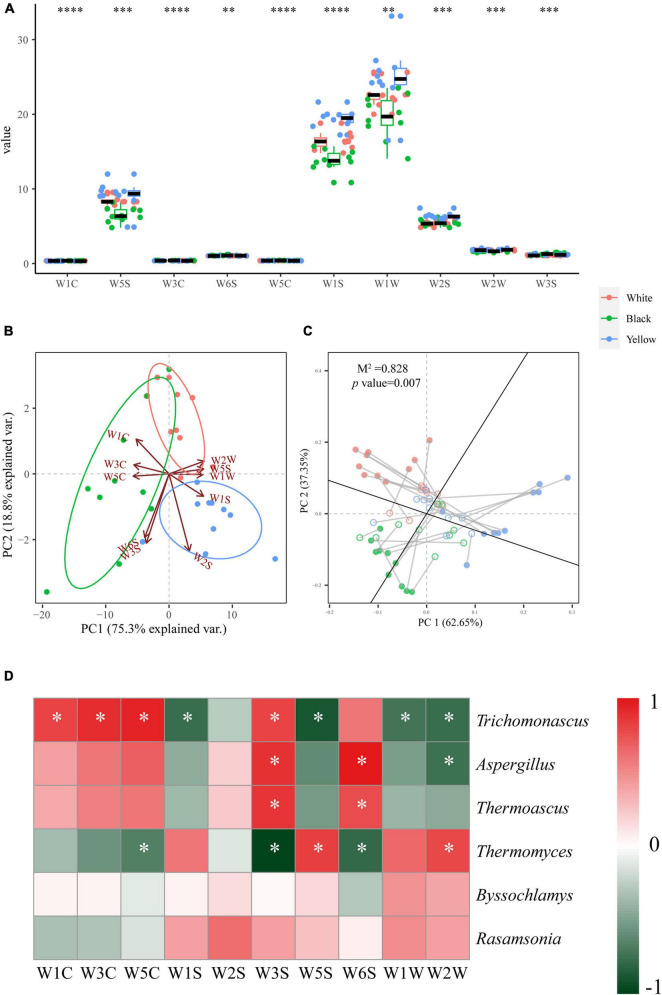
Box plot for aroma profiles of the three types of HTD samples **(A)**. Significant difference is represented by ^****^ (*p* < 0.0001), ^***^ (0.0001 ≤ *p* < 0.001), and ^**^ (0.001 ≤ *p* < 0.01), respectively. PCA biplot based on the aroma profiles of HTD samples **(B)**. Procrustes analysis of the correlation between dominate fungal genera and aroma profiles (*M*^2^ = 0.828, *p* = 0.007, 999 permutations) **(C)**. Heatmap depicting the Spearman’s rank correlation between dominant fungal genera and E-nose sensors. Significant difference is represented by * (*p* < 0.05) **(D)**.

The aroma of HTD samples was subsequently determined using E-nose (Figures 6A,B). As illustrated in Figure 6A, although all 10 E-nose sensors also exhibited extremely significant difference, only the WC sensors (W1C, W3C, and W5C) for aromatic compounds and W1S sensor for methane differed to a level of *p* < 0.0001 (Cai et al., 2019, 2020b). Once again, PCA was carried out to visualize the aroma profiles of different types of HTD on the basis of E-tongue data (Figure 6B). PC1 and PC2 accounted for 75.3 and 18.8% of the variance for PC1 and PC2, respectively. These led to total variance of 94.1% altogether. It is known from Figure 6B that, except for a few samples, the three types of HTD showed a clear separation trend. White HTD and yellow HTD that mainly situated to the right of the biplot were discriminated by W1S sensor for methane, W2S sensor for alcohols and ketones, W5S sensor for nitrogen oxides, and WW sensors (W1W and W2W) for sulfur organic compounds, whereas black HTD lying on the left of the biplot were mostly differentiated mainly by W3S for methane-aliph, W6S for hydrogen, and WC (W1C, W3C, and W5C) sensors for aromatic compounds (Cai et al., 2019, 2020b). Further, Procrustes analysis revealed an extremely significant correlation between the dominant fungal genera and aroma profiles of HTD samples (*M*^2^ = 0.828, *p* = 0.007 < 0.01) (Figure 6C). Hence, combined with the results of taste analysis of HTD samples based on E-senses (Figure 5), the fungal communities in HTD mainly affect the aroma rather than taste.

To clarify the specific effect of the fungal communities in HTD on the aroma, the Spearman’s correlation coefficient between the dominant fungal genera and the E-nose sensors was calculated, and the results were depicted using a heatmap (Figure 6D). A total of 18 significant correlations were found between the six dominant fungal genera and the 10 E-nose sensors (*p* < 0.05). Among which, *Trichomonascus* was positively correlated with WC sensors (W1C, W3C, and W5C, for aromatic compounds) and W3S sensor (for methane-aliph) and negatively correlated with W1S sensor (for methane), W5S sensor (for nitrogen oxides), and WW sensors (W1W and W2W, for sulfur organic compounds); *Aspergillus* was positively correlated with W3S sensor for methane-aliph and W6S sensor for hydrogen and negatively correlated with W2W sensor (for aromatic compounds and sulfur organic compounds); *Thermoascus* was positively correlated with W3S sensor (for methane-aliph) and W6S sensor (for hydrogen); whereas *Thermomyces* was positively correlated with W5S sensor (for nitrogen oxides) and W2W sensor (for aromatic compounds and sulfur organic compounds) and negatively correlated with W3S sensor (for methane-aliph), W6S sensor (for hydrogen), and W5C sensor (for alkanes and aromatic compounds).

## Discussion

High-temperature Daqu is an essential saccharifying starter in the fermentation of SFB (Li et al., 2017; Yang et al., 2018). It is not only a major source of microorganisms and an important crude enzyme preparation but it also provides the basis for brewing materials and flavor precursors contributing to the formation of the sauce flavor characteristics of the final products. Presently, the understanding of different types of HTD is only on the difference of appearance color, which is subjective and unscientific. Not long ago, an analysis of the bacterial diversity and functional divergences in the different types of HTD by our team found that, although the three types of HTD had similar functions and phenotypes, the bacterial communities differed significantly (*p* < 0.05) (Wang Y. R. et al., 2021). However, the fungal diversity and flavor profiles of the three types of HTD have yet to be resolved. Therefore, fully understanding the fungal communities and flavor might lead to a more comprehensive assessment on the applicability of HTD for liquor brewing.

Li et al. (2014) reported that the black HTD has almost no yeasts and molds – only bacteria, adapted to the unique environment of high temperature and hypoxia, are able to survive. Inconsistent with their report, in the present study, abundant fungi were detected not only from black HTD but also from white HTD and yellow HTD. *Thermomyces*, *Thermoascus*, *Aspergillus*, *Rasamsonia*, *Byssochlamys*, and *Trichomonascus* were observed to be the dominant fungal genera in HTD (Figure 2). Different from the observations of the present study, Wang et al. (2019) combined culture-dependent with culture-independent methods and revealed that dominant fungi in HTD from Moutai town, Guizhou Province, China, were *Aspergillus Rhizopus* and *Saccharomycopsis*. This suggests that environmental differences contribute to the divergences in fungal communities HTD from different regions.

When Wang et al. (2008) explored the fungi in HTD by culture-dependent method, they found that the fungi in HTD were mainly consisted of molds including *Aspergillus*, *Mucor*, *Rhizopus*, *Monascus*, and *Trichoderma* along with yeasts comprising *Saccharomyces*, *Hansenula*, *Candida*, *Pichia*, and *Torulaspora*. However, in this study, using high-throughput sequencing technology, thermophilic fungi, such as *Thermomyces*, *Thermoascus*, and *Byssochlamys* with higher relative abundance, were found in HTD, which was different from the results obtained by culture-dependent method but in line with the assertion obtained by Hu et al. (2017) through culture-independent method. These thermophilic fungi rarely appeared in the previous literatures of fungal communities in HTD by culture-dependent method and are generally considered as non-dominant microorganisms. Because comparing with other microorganisms, their growth requires specific culture conditions. Among the six absolute dominant fungal genera in the present study, *Thermomyces*, *Thermoascus*, and *Byssochlamys* are thermophilic fungi that can grow and metabolize under high temperature environment, so these genera are abundant in HTD. In particular, *Byssochlamys*, which produce heat-resistant ascospores, can survive for a considerable period of time at temperatures above 85°C and grow under very low oxygen tension (Houbraken et al., 2006). This may also explain why the relative abundance of *Byssochlamys* was highest in black HTD (distributed in the central part of the Daqu piles with the worst air permeability and the least oxygen contact), whereas lowest in white HTD (distributed in the top layer of Daqu piles and is more sufficiently exposed to oxygen), and the difference was significant (*p* < 0.05) (Figure 2; Wang Y. R. et al., 2021). These thermophilic fungi are closely related to cellulose degrading enzymes (cellulase and glucosidase, etc.) and starch hydrolase (glucose) synthesis (Thorsen et al., 2006; Schuerg et al., 2017; Timo et al., 2017).

Moreover, three core OTUs were found in all 30 HTD samples, and two of them were affiliated to *Aspergillus* (Figure 3B), which is also the absolute dominant fungal genera in HTD. *Aspergillus* has been detected as the dominant mold in low, medium, and high temperature Daqu and produces a wide range of enzymes and metabolites (Hu et al., 2017; Fan et al., 2018; Du et al., 2019; Shoubao et al., 2019; Wang et al., 2019). This genus can secrete various saccharifying hydrolases into its environment, which degrade and convert starch into sugars, to further promote the growth, reproduction, and metabolism of bacteria and yeasts (Masayuki et al., 2008; Shoubao et al., 2019). It also produces proteolytic along with other lytic enzymes that contribute flavonoid formation and protein hydrolysis (Masayuki et al., 2008; Gou et al., 2015; He et al., 2019). Meanwhile, *Aspergillus* is considered as the key to the sauce flavor formation, because it has positive correlations with pyrazines, esters, and certain aromatics (Jin et al., 2019).

Both PCoA (Figure 4A) and MANOVA (Figure 4B) based on Bray–Curtis distance demonstrated that the fungal communities of the three types of HTD differed significantly (*p* < 0.05), and the fungal communities of black HTD samples and yellow HTD samples bear a stronger resemblance than that of white HTD samples. Further comparison of the within-group variations of the three types of HTD revealed that black HTD samples exhibited the highest within-group variation among the three types of HTD. The reason may be related to the temperature distribution in the fermentation chamber during the HTD-making process, because the piling process of HTD leads to the difficult loss of water between the HTD (white HTD samples and black HTD) in the middle of the fermentation chamber, the faster temperature rise of HTD, and the longer duration; whereas the temperature in other parts of the fermentation chamber is lower than the temperature in the middle of the fermentation chamber, thus resulting in variation of fungal communities within different groups of HTD.

According to the results of LEfSe (Figures 4D,E), *Thermomyces* was demonstrated to be the biomarker in white HTD. *Thermomyces* is the absolute dominant fungus during the fermentation of both Chinese SFB and light-flavor Baijiu (Shi et al., 2010). It has good thermal stability and high viability in a variety of adverse environments and could survive at temperatures above 60°C (Singh et al., 2003; Zheng et al., 2015). *Thermomyces* is also the source of important enzymes in Chinese Baijiu fermentation and can produce cellulase and protease with high vitality and thermal stability to promote the degradation of macromolecular polysaccharides along with proteins. It is beneficial not only to the utilization of Chinese Baijiu–making materials by microorganisms but also to the promotion of microbial growth and Chinese Baijiu flavor production (Zheng X. et al., 2012; Jin et al., 2019).

The biomarker in black HTD was *Thermoascus*. It can grow and metabolize in high temperature environment above 45°C and produce enzymes with high thermal stability and activity, such as catalase (removing cytotoxic substances such as hydrogen peroxide), endoglucanase (one of the main components of cellulase system), glucosidase (breaking down glucoside bonds, which is one of the important enzymes in microbial metabolism), keratinase (the food industry has catalytic esterification), chitinase (hydrolyzing chitin, inhibiting fungal pathogens), and other enzymes (Boyce and Walsh, 2021). These enzymes are able to degrade starch or cellulose from raw materials during the process of Chinese Baijiu brewing, making *Thermoascus* an indispensable and important fungal genus in HTD (Jain et al., 2015).

*Monascus* was the biomarker in yellow HTD. It is the main mold in the brewing of SFB and has strong saccharifying and fermenting power and esterification power (Wang et al., 2008; Xu et al., 2021a). *Monascus* can also secrete esterase, amylase, and acid protease, which is crucial to the high temperature and acidic environment of Chinese Baijiu making, and can also produce saccharifying enzymes with higher activity to improve the yield of Chinese Baijiu and produce more ester compounds (Chen et al., 2011; Xu et al., 2021b). At the same time, it also produces a variety of secondary metabolites beneficial to human body, such as masculin, gamma-aminobutyric acid, and other functional components, which improve the nutritional and health value of Chinese Baijiu (Dikshit and Tallapragada, 2015; Yun-lei et al., 2015; Xu et al., 2021b).

Flavor is one of the most decisive features for characterizing foods, including two aspects: taste and aroma (Cai et al., 2019; Guo et al., 2019). HTD is not only a saccharifying starter but also a partial source of raw materials for SFB making. Therefore, it is necessary to evaluate the overall flavor of HTD samples with E-tongue and E-nose and to explore the relationship of the dominant fungal genera and flavor. All eight tastes indicators of the three types of HTD were extremely significantly different (*p* < 0.0001), but the differences in the response values of aftertaste-A and aftertaste-B were less than 1 (Figure 5A). This suggests that, except for the aftertaste of astringency and bitterness, other taste differences can be distinguished by the human tongue and affect the consumers’ preferences (Cai et al., 2020a). Thus, the taste differences were most pronounced in white HTD characterized by sourness, bitterness, astringency, and richness and in yellow HTD characterized by saltiness and umami, and the taste characteristics of black HTD characterized by aftertaste-A and aftertaste-B were not prominent (Figures 5A,B). Although the three types of HTD tastes different, their taste is minimally influenced by fungal communities (Figure 5C).

In terms of the aroma in the three types of HTD, the differences in response values of WC sensors (W1C, W3C, and W5C) for aromatic compounds and W1S sensor for methane were the most significant, reaching the same level of *p* < 0.0001 as the eight tastes indicators determined by E-tongue (Figure 6A). This indicates that different types of HTD differed most significantly in taste, aromatic compounds, and methane. The three types of HTD have their own unique aroma characteristics: methane-aliph, hydrogen, and aromatic compounds were enriched in black HTD, whereas methane, alcohols, ketones, nitrogen oxides, and sulfur organic compounds were enriched in white HTD and yellow HTD. Procrustes analysis demonstrated that fungal communities were responsible for the differences in aroma characteristics of the three types of HTD (*M*^2^ = 0.828, *p* = 0.007 < 0.01) (Figure 6C). This is in accordance with the observation of Cai et al. (2021b) in low temperature Daqu that the aroma of Daqu was mainly affected by fungi, indicating that, regardless of the temperature during Daqu making, its aroma was largely contributed by fungi. Correlation analysis further clarified the specific impact of the dominant fungal genera on aroma (Figure 6D): *Trichomonascus*, which is significantly enriched (*p* < 0.05) in black HTD, can elevate the levels of aromatic compounds and methane-aliph and inhibit the generation of methane, nitrogen oxides, and sulfur organic compounds; *Aspergillus*, which is significantly enriched (*p* < 0.05) in black HTD and yellow HTD, and *Thermoascus*, which is significantly enriched (*p* < 0.05) in black HTD, can both elevate the levels methane-aliph and hydrogen, but *Aspergillus* also inhibits the generation of aromatic compounds and sulfur organic compounds; whereas *Thermomyces*, which is significantly enriched (*p* < 0.05) in white HTD, can elevate the levels of nitrogen oxides, aromatic compounds, and sulfur organic compounds and inhibit the generation of methane-aliph, hydrogen, alkanes, and certain aromatic compound. Therefore, *Trichomonascus*, *Aspergillus*, *Thermoascus*, and *Thermomyces* were the fungal genera that contribute the most to the aroma in HTD, and with their combined effect, the three types of HTD generated their respective unique aroma profiles.

Aromatic compounds and sulfur organic compounds are the two most widely reported classes of aroma compounds in Baijiu: Aromatic compounds were among the skeleton aroma compounds that contributed most to the overall flavor of Chinese Baijiu (He et al., 2019), whereas sulfur organic compounds are important aroma compounds in various fermented foods and alcoholic beverages with low detection thresholds and unpleasant aroma of onion and cooked cabbage (Cai et al., 2020b; Zhao et al., 2020). *Trichomonascus*, *Aspergillus*, and *Thermomyces*, which were enriched in the three types of HTD, respectively, all showed strong correlations aromatic compounds and sulfur organic compounds, suggesting each type of HTD contributed to the aroma of SFB, but their metabolic mechanism awaits further investigation.

## Conclusion

The results of the present study showed that ITS high throughput sequencing was effective to access the fungal communities of HTD, and the fungal communities in HTD are significantly diverse (*p* < 0.05). Six fungal phyla along with 54 fungal genera were detected from 30 HTD samples. Ascomycota and Basidiomycota were absolute dominant fungal phylum in the three types of HTD samples, whereas *Thermomyces*, *Thermoascus*, *Aspergillus*, *Rasamsonia*, *Byssochlamys*, and *Trichomonascus* were dominant genera. The three types of HTD each had a unique flavor profile, and the differences on aroma were mainly influenced by the dominant fungal genera including *Trichomonascus*, *Aspergillus*, *Thermoascus*, and *Thermomyces*. Each type of HTD contributed to the aroma of SFB; however, the exact relationships and mechanisms of these fungal communities and flavor compounds in the three types of HTD remain to be further explored and clarified. This study provides insight into the fungal diversity of different types of HTD and sets the stage for future studies on the flavor formation mechanism in SFB and the improvement of HTD quality. On the basis of these results, we can further select functional microorganisms related to the performance of HTD.

## Data Availability Statement

The datasets presented in this study can be found in online repositories. The names of the repository/repositories and accession number(s) can be found below: https://www.ncbi.nlm.nih.gov/, PRJNA773630.

## Author Contributions

WC: software, formal analysis, writing – original draft, writing – review and editing, and visualization. YX: investigation, validation, and data curation. YW: investigation and validation. WW: resources and conceptualization. NS: investigation and data curation. HZ: resources and validation. FT: project administration. XY: resources and supervision. CS: project administration and supervision. ZG: conceptualization and funding acquisition. All authors contributed to the article and approved the submitted version.

## Conflict of Interest

The authors declare that the research was conducted in the absence of any commercial or financial relationships that could be construed as a potential conflict of interest.

## Publisher’s Note

All claims expressed in this article are solely those of the authors and do not necessarily represent those of their affiliated organizations, or those of the publisher, the editors and the reviewers. Any product that may be evaluated in this article, or claim that may be made by its manufacturer, is not guaranteed or endorsed by the publisher.

## Footnotes

1
https://www.r-project.org/

2
https://www.python.org/

## Supplementary Material

Supplementary Figure 1 Rarefaction **(A)** and Shannon diversity **(B)** curves of the three types of HTD samples.
