# Supplementary figures and images for: The Fungal Communities and Flavor Profiles in Different Types of High-Temperature Daqu as Revealed by High-Throughput Sequencing and Electronic Senses
Source: Front Microbiol. 2021 Dec 2;12:784651. doi: 10.3389/fmicb.2021.784651 (PMC8674350; doi:10.3389/fmicb.2021.784651)

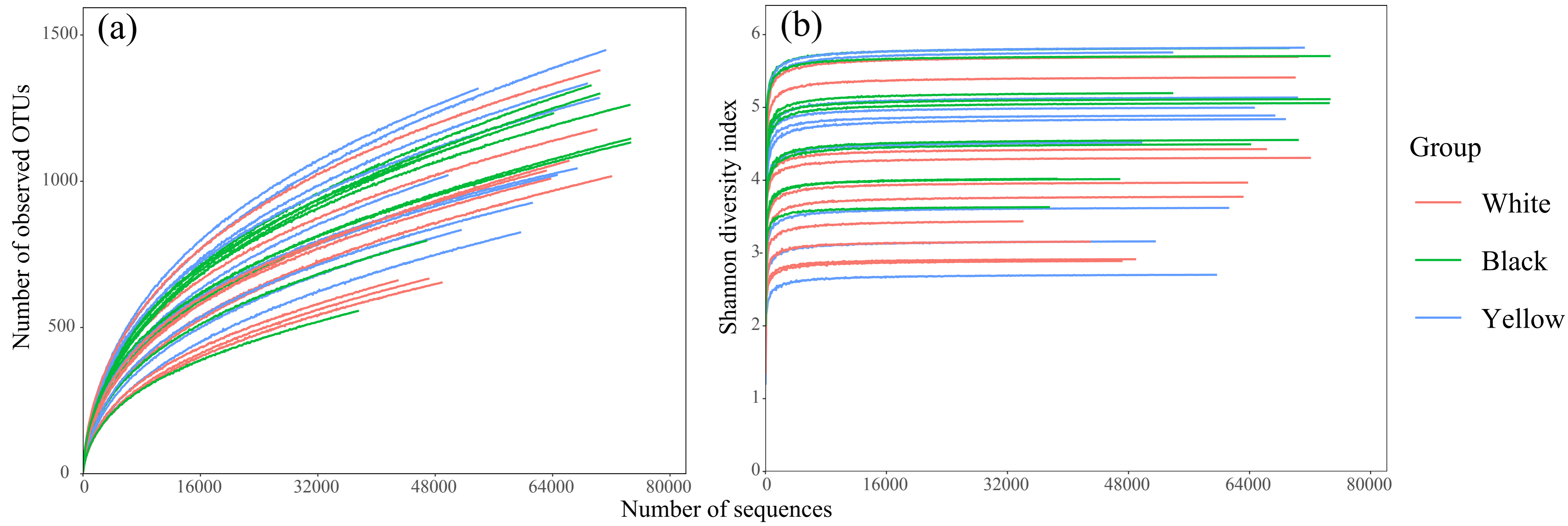

Supplement: Supplementary Figure 1 — Rarefaction (A) and Shannon diversity (B) curves of the three types of HTD samples. [file Image_1.pdf]
